# Effectiveness of Reminiscence Therapy versus Cognitive Stimulation Therapy in Older Adults with Cognitive Decline: A Quasi-Experimental Pilot Study

**DOI:** 10.3390/nursrep12020033

**Published:** 2022-05-02

**Authors:** Isabel Gil, Paulo Santos-Costa, Elzbieta Bobrowicz-Campos, Rosa Silva, Maria de Lurdes Almeida, João Apóstolo

**Affiliations:** 1 Health Sciences Research Unit: Nursing (UICISA: E), Nursing School of Coimbra (ESEnfC), 3046-851 Coimbra, Portugal; paulocosta@esenfc.pt (P.S.-C.); rcgsilva@esenfc.pt (R.S.); mlurdes@esenfc.pt (M.d.L.A.); apostolo@esenfc.pt (J.A.); 2 Instituto Ciências da Saúde, Universidade Católica Portuguesa, 4169-005 Porto, Portugal; 3 Faculty of Psychology, Education Sciences of the University of Coimbra, 3001-802 Coimbra, Portugal; elzbieta.campos@gmail.com

**Keywords:** older adults, cognitive decline, reminiscence therapy, cognitive stimulation, depressive symptomatology, quality of life

## Abstract

Cognitive impairment can represent a predecessor to neuro-degenerative processes; however, evidence suggests that non-pharmacologic interventions such as reminiscence therapy (RT) and cognitive stimulation therapy (CST) can potentially stabilize or reverse this trend. Community-based settings are widely regarded as the key area of intervention by healthcare professionals in this field. Thus, this study aimed to assess the effects of an RT and a CST program in the cognition, depressive symptomatology, and quality of life (QoL) of older adults with cognitive decline who attend community support structures (CSS) in central Portugal. A quasi-experimental study with two arms (RT and CST program) was conducted for seven weeks. Participants were allocated to each arm based on the CSS they attended. Of the 109 older adults initially screened, 76 completed the intervention (50 in the RT program and 26 in the CST program). A pre- and post-intervention analysis showed statistically significant differences in older adults’ cognition, especially in their delayed recall ability, in both groups. Older adults in the RT program evidence improved QoL scores post-intervention. Both the RT and CST programs implemented throughout the study are beneficial to older adults’ cognitive performance, although results are more pronounced in the earlier stages of cognitive decline. Participation in the RT program was associated with improvements in older adults’ QoL scores.

## 1. Introduction

Living longer is associated with greater risks of social isolation and loneliness, as well as physical, mental, and economic dependence, often resulting in the stigmatization of older adults [1]. Thus, in the last few decades, clinicians and researchers have delved into older adults’ cognitive frailty as a focus of increasing societal importance. Studies substantiate the association between cognitive and physical frailty, marked by a progressive decline in intellectual performance [2], especially in older adults’ attention, executive function, and processing speed [3].

Although cognitive frailty is recognized as a precursor to neurodegenerative conditions, early identification and intervention can potentially stabilize, or reverse, this process [2,4]. Parallel to this challenge, the World Health Organization’s Action Plan for 2013–2020 highlights the importance of providing comprehensive healthcare in community and primary settings, preserving older adults’ self-care and well-being, and reducing the need for their institutionalization [5]. This challenge warrants new reforms in the healthcare and social systems, through the implementation and development of community-based psychosocial interventions involving older adults, as well as their relatives and significant others.

Several strategies have been described extensively in the literature to prevent physical frailty [6], such as exercise routines and nutritional supplementation. Similarly, the literature often suggests that early implementation of non-pharmacologic interventions such as cognitive stimulation therapy (CST) [7,8,9] and reminiscence therapy (RT) [10,11] can preserve older adults’ cognitive function. CST is characterized by a set of significant activities, developed over several sessions that usually take place in a social context, and which aim to stimulate several domains, including attention, memory, language, and calculation, among others [7]. CST enhances cognitive performance and reduces depression in older people, while also showing promise in terms of improving their quality of life (QoL). Another advantage of CST is its ability to be used with people from various socioeconomic and cultural backgrounds [12,13]. RT promotes the older person’s self-esteem, identity, and uniqueness by reuniting them with major life experiences and resolving self-conflicts via enjoyable and stimulating activities [10]. Despite the fact that certain studies have shown that RT can improve people’s quality of life (QoL), cognition, and mood, the effects are inconsistent, modest in size, and vary greatly among situations and modalities [14].

Few studies, however, have directly examined the potential therapeutic effects of CST and RT in cognitively frail older adults, highlighting the need for further research in this field, particularly when used in community-based settings [7,15]. Thus, this study aims to assess the effects of an RT and a CST program on cognition, depressive symptomatology, and QoL in older adults with cognitive frailty, who attend community support structures.

## 2. Materials and Methods

### 2.1. Study Design

Between April and June 2017, a pilot quasi-experimental study was carried out for seven weeks with two intervention groups (CST versus RT). The research team consisted of healthcare professionals and researchers with substantial experience in the delivery of CST and RT sessions. Study participants were recruited from seven community support structures (CSS) in the central region of Portugal.

### 2.2. Procedures

Formal authorization was granted by the community support structures before study commencement. The research team met locally with representatives from each CSS and presented the study’s aim and details (e.g., inclusion/exclusion criteria, type of interventions planned, and assessment periods). Each CSS introduced the study to the older adults’ attending their services and provided contact details to the research team. At each institutional visit, the research team approached the older adults, provided further details about the study, and obtained their informed consent. Study implementation and data collection were performed at each CSS.

### 2.3. Intervention Programs

Both intervention programs were composed of 14 sessions, held twice a week, with a duration of 45 to 60 min each. The sessions were developed as group-based interventions, facilitated by a leader and a co-leader from each CSS. The selected leaders had to: (i) be a certified professional working in each CSS (e.g., nurses, gerontologists, and social service workers); and (ii) attend training sessions provided by the research team before study commencement. During the training sessions, leaders were given the manuals from each intervention, with a detailed list of the required resources and complementary activities. While the first session was conducted by the research team (with a non-participative observation by the leaders), the following sessions were conducted by the leaders with supervision. After each session, feedback was exchanged by the research team and leaders.

Given the combined training sessions, those administering the intervention were not blinded to treatment allocation, but were strongly inculcated not to disclose this information at the follow up assessments.

The CS intervention was based on the “Making a Difference” program, specifically developed for older adults with cognitive decline, and previously adapted and validated to the European Portuguese language and culture [7,15]. This program offers a sequence of activities that covers different cognitive domains and promotes older adults’ socialization and self-esteem. The RT program is composed of activities that follow older adults’ lifespan (e.g., school, professional life, travelling, holidays and celebrations, and historical dates/moments). Such activities allow older adults to revive and share life-changing/significant moments and integrate them into their autobiographical narrative. The program was developed and validated by Gil and colleagues [16]. Both interventions took place in CSS-provided rooms that were spacious and well-equipped (usually used by the institutional care teams for other group dynamics and activities).

### 2.4. Participants

To be considered for study inclusion, participants had to: (i) be 65 years of age or older; (ii) have the ability to give informed consent before study commencement; (iii) have the ability to participate in group activities for a period between 45 to 60 min; (iv) have no pronounced impairment of their visual and auditory abilities; and (v) have mild to moderate cognitive decline, assessed as a score equal to or below 20 points in the Six-Item Cognitive Impairment Test (6-CIT) [17]. Older adults with an unstable clinical condition or those prescribed with cholinesterase inhibitors, and/or memantine, and/or antipsychotics during the study period were excluded.

The initial sample was composed of 150 older adults (116 female and 34 male). After an initial assessment of the inclusion criteria, 109 older adults were included in the study. Allocation to each intervention was performed according to the older adults’ CSS, with RT being implemented in four institutions and CS in three. Thus, older adults were unaware of treatment assignment. All older adults that did not meet inclusion criteria were offered the opportunity to participate in thematic group discussions developed in each CSS by the local leaders.

### 2.5. Assessment and Instruments

Data were collected during the participants’ eligibility screening (T0), initial assessment (T1), and final assessment (T2). At T0, data were first collected through a semi-structured interview focused on the participant’s clinical history, conditions, and current prescriptions. Then, three scales were used (culturally adapted and validated for the Portuguese population): (i) the 6-CIT [17]; (ii) the Lawton Instrumental Activities of Daily Living (IADL) Scale [18]; and (iii) the Tilburg Frailty Indicator (TFI) [19]. The outcomes of interest for T1 and T2 included cognition, depressive symptomatology, and QoL, assessed through the culturally adapted versions of the Montreal Cognitive Assessment (MoCA) [20], the 10-items Geriatric Depression Scale (GDS-10) [21], and the 8-items version of the World Health Organization Quality of Life—module for older adults (WHOQOL-OLD-8) [22,23]. Data collection was performed by members of the research team without previous contact and information on which intervention was implemented in each CSS.

### 2.6. Ethical Considerations and Procedures

This study complied with the ethical principles for medical research involving human subjects, as per the Declaration of Helsinki. Before study commencement, approval was received by the Ethics Committee of the Health Sciences Research Unit: Nursing from the Nursing School of Coimbra (Ref. P406-03/2017). The study protocol was registered a posteriori in clinicaltrials.gov (identifier NCT05187572), although no deviations occurred from the initial study protocol submitted to the Ethics Committee. The study adheres to the Transparent Reporting of Evaluations with Nonrandomized Designs (TREND) reporting statement.

### 2.7. Data Analysis

Statistical analysis was performed using the SPSS Statistics program (version 24, IBM SPSS, New York, NY, USA). Descriptive statistics of central tendency and dispersion, frequencies, and percentages were calculated for sociodemographic variables and for the clinical indicators measured at different assessment times (pre- and post-intervention). For the inferential analysis, the level of significance was assumed with the probability of a type I error of 5% (*p* = 0.05). To compare the distribution of continuous and categorical variables between groups, the Mann–Whitney and Chi-Square non-parametric tests were used, respectively, because they did not meet the assumptions that support the use of parametric tests (e.g., normality of distribution).

The Wilcoxon test was used to assess the impact of the RT and CST programs on cognition, depressive symptomatology, and QoL. The effect size was estimated according to the formula r = Z/√N [24,25]. Associations between continuous variables were analyzed using Spearman’s correlation coefficient (rho). Weak associations were considered for coefficients less than ±0.4, moderate associations for coefficients ranging between ±0.4 and ±0.69, and strong associations for coefficients ranging between ±0.7 and ±0.89. The remaining values (equal to or greater than ±0.9) were considered as indicators of very strong associations [26].

## 3. Results

Of the 150 participants included in the screening assessment, 41 were excluded for not meeting the inclusion criteria. The reasons given for the participants’ exclusion were: 6-CIT score > 20 (*n* = 18); taking anti-psychotics/cholinesterase (*n* = 7); unable to remain in a group intervention (*n* = 5); sensory/communicational impairment (*n* = 4), age < 65 years (*n* = 3); refusal to participate/withdrawal (*n* = 3); and unstable condition (*n* = 1).

### 3.1. Participants’ Characterization

The Mann–Whitney test indicated that the participants in both programs, pre-intervention, did not differ significantly in terms of age, education, frailty status, overall ability to perform instrumental activities of daily living, and QoL (*p* > 0.05) (Table 1).

Using the 6-CIT test, statistically significant differences were found between participants of the RT and CST programs, with the older adults allocated to the latter evidencing higher scores. However, such a difference between groups was not verified when analyzing the scores obtained in the MoCA test.

Of the 109 older adults who participated in the screening assessment (T0), only 76 completed the program; 50 in the RT program and 26 in the CST program. Most of the dropouts were due to worsening of the clinical condition and/or need for hospitalization (*n* = 30), personal reasons (*n* = 2), and undisclosed reasons (*n* = 1). For the remaining participants, between T1 and T2, no changes of clinical status or medication were recorded.

### 3.2. Impact of the RT and CST Programs in Cognition, Depressive Symptomatology, and QoL

Concerning older adults’ cognition, within-group analysis of the MoCA global scores revealed statistically significant differences pre- and post-intervention in the RT group (*p* = 0.005) with a small effect size (r = 0.398). However, only a marginally significant difference was found in the CST group (*p* = 0.076), with a small effect size (r = 0.348). The cognitive domain that mostly improved post-intervention in the RT group was delayed recall (*p* = 0.005), with a small effect size (r = 0.397). Additionally, older adults in the RT group also evidenced marginally significant improvements in the MoCA’s naming domain (*p* = 0.077), with a small effect size (r = 0.250). Interestingly, older adults in the CST program also evidenced higher scores of delayed recall post-intervention (*p* = 0.021), although with a small effect size (r = 0.451).

Moreover, a statistically significant improvement of older adults’ QoL scores was evidenced in the RT program (*p* = 0.009), with a small effect size (r = 0.369). Although in both groups the global GDS-10 scores decreased pre- and post-intervention, the effect was not statistically significant (Table 2).

The number of older adults who improved, worsened, and maintained their cognitive function, depressive symptomatology, and QoL is presented in Table 3. RT showed a marginally significant impact on the reduction of the number of older adults with depressive symptomatology (46%) when compared to CST (27%). However, no statistically significant difference between groups was observed for cognition and QoL. 

### 3.3. Correlations between Clinical and Sociodemographic Variables and Changes in the Outcomes of Interest

A correlation analysis was performed, considering older adults’ age, educational level, frailty status, performance in IADL, and differences in the MoCA, GDS-10, and WHOQOL-OLD-8 scores pre- and post-intervention. In the RT group, no statistically significant correlation was found; however, a moderate, statistically significant negative correlation was found between the WHOQOL-OLD-8 and the GDS-10 scores (rho = −0.515, *p* = 0.002). Concerning the CST group, a significant and moderate correlation was observed between older adults’ age and the differences observed pre- and post-intervention in the global MoCA scores (rho = −0.414, *p* = 0.036). Moreover, a strong, statistically significant negative correlation was observed between the WHOQOL-OLD-8 and GDS-10 scores (rho = −0.721, *p* = 0.002). No other statistically significant correlations were observed in both groups.

## 4. Discussion

Both interventions revealed a positive effect on older adults’ cognition, with significant improvements in their delayed recall ability. In the RT group, marginally significant improvements were also observed in the older adults’ naming domain. These results converge with the findings of recent systematic reviews, which highlighted the effectiveness of RT in improving cognitive functions in older adults with cognitive decline [11,14,27]. Concerning CST, several studies found evidence of its therapeutic effect in several cognitive domains, such as language, memory, and learning ability [27,28,29]. Interestingly, Macedo and colleagues [29] highlight that the therapeutic effects of CST programs are persistently more pronounced in older adults who live with their relatives when compared with those who are institutionalized. This might be explained by a greater involvement from and support by older adults’ significant others, as well as more availability and resources to provide cognitive and sensorial stimulation in community-based settings in comparison with geriatric institutions, where the professionals’ attention is divided between a higher number of people and with a focus on a more diverse set of care activities. Both RT and CST are based on sensory and cognitive stimulation through engaging and intrinsically satisfying activities that promote neuroplasticity [30]. Thus, RT and CST can actively delay the clinical manifestations of dementia in older adults.

In the CST group, a significant association, with moderate magnitude, was observed between age and differences in the MoCA global scores; that is, as age advances, the effect of the program on cognition is lower. This result highlights the importance of implementing CST interventions in the earlier stages of ageing, thereby enhancing its potential in preventing cognitive decline.

Recent systematic reviews emphasized the inconsistent results found in the literature regarding the effect of RT in reducing depressive symptomatology, even in community-based settings [11,14]. Accordingly, in our study, although the average scores in the GDS-10 lowered between T1 and T2, no statistically significant results were observed that corroborate the therapeutic potential of RT and CST in reducing older adults’ depressive symptomatology. We suggest that future studies in this field include more comprehensive scales, specifically validated to the older population with cognitive decline, as well as a qualitative assessment of older adults’ depressive symptomatology pre- and post-intervention.

Regarding QoL, in this study, a statistically significant improvement was found solely in the RT group. Our results mirror the findings by Nakatsuka and colleagues [31], who found improvements in the RT group. Nonetheless, contrary to the findings of Nakatsuka and colleagues, no improvements in the older adults’ QoL were found in the CST group in this study.

Some limitations must be addressed. First, the loss of 33 participants pre- and post-intervention may hinder the analysis of both programs’ true effects on older adults’ cognition, depressive symptomatology, and QoL. Moreover, the taking of antidepressants was not considered an exclusion criterion, and the exclusion of older adults who were taking cholinesterase inhibitors, and/or memantine, which may explain the inconsistent results found in this study. Another aspect that may be a limitation is the inclusion of older adults with and without symptoms of depression, which may also have influenced the results. The small sample size, especially when analyzing the number of participants per group, may have conditioned the assessment of the RT and CST programs’ effectiveness on the outcomes of interest. Future studies must address such limitations to perform a comprehensive assessment of the selected interventions. Finally, due to human error, the study protocol was not made available online for public consultation before study commencement. The lack of a public protocol can lead to the selective reporting of study outcomes by its authors (outcome reporting bias), potentially compromising the validity of an experiment and any subsequent meta-analysis. In this study, although a protocol was registered posteriorly, this is unideal. Future authors must assure that this step is performed, maintaining a policy of transparency in research.

Nonetheless, to the best of the authors’ knowledge, this study was the first in Portugal to assess the effectiveness of a structured RT group program in the cognition, depressive symptomatology, and QoL of older adults with cognitive decline. Furthermore, no previous studies are known that compared the effectiveness of both interventions in Portuguese older adults with cognitive decline that attended CSS. Given that in Portugal, until now, there were no investigations that focused on the effectiveness of a structured group RT program aimed at older people with cognitive decline, in the context of community support structures, this pilot study is a starting point for carrying out further investigations with larger samples and more robust designs, such as randomized controlled studies. Despite the limitations identified, the results presented support the importance of implementing non-pharmacologic interventions such as RT and CST in CSS attended by older adults with cognitive decline.

## 5. Conclusions

The RT and CST programs implemented in this study proved to be effective in improving older adults’ cognition, especially in the earlier stages of cognitive decline. Regarding depressive symptomatology, although a positive trend was observed in the average scores of the older adults who attended the RT program, this was not statistically significant. However, a statistically significant improvement of older adults’ QoL scores was evidenced in the RT program, with a small effect size. No statistically significant differences were found in older adults attending the CST program regarding depressive symptomatology or QoL.

## Figures and Tables

**Table 1:** Characterization of the participants who integrated the T1 assessment in the RT and CST groups (*n* = 109).

	RT (*n* = 76)	CST (*n* = 33)	*p*
**Sex** (female/male)	57/19	28/5	0.254
**Age** (years) M (±SD; min–max)	79.65 (±7.08; 65–93)	81.18 (±8.85; 66–99)	0.355
**Education** (years) M (±SD; min–max)	4.00 (±3.00; 0–12)	4.00 (±3.00; 0–12)	0.514
**IADL**: M (±SD min–max)	15.25 (±7.06; 0–23)	14.27 (±6.40; 2–23)	0.314
**6-CIT**: M (±SD; min–max)	8.07 (±5.92; 0–20)	10.67 (±5.25; 0–20)	**0.031** *
**TFI**: M (±SD; min–max)	6.89 (±2.57; 1–12)	7.52 (±2.45; 3–11)	0.306
**GDS-10**: M (±SD; min–max)	3.55 (±2.90; 0–10)	4.39 (±3.45; 0–10)	0.413
**WHOQOL-OLD-8**: M (±SD; min–max)	27.70 (±4.23; 14–38)	25.61 (±4.83; 17–33)	0.081
**MoCA**: M (±SD; min–max)	14.11 (±5.67; 3–24)	13.70 (±4.24; 4–22)	0.641

**M**: mean; **SD**: standard deviation; **min**: minimum; **max**: maximum; * statistically significant difference (*p* < 0.05; Mann–Whitney test).

**Table 2:** Effect of the RT and CST programs in the outcomes of interest.

	RT (*n* = 50)			CST (*n* = 26)		
	T1	T2	*p*	T1	T2	*p*
**GDS-10** M (±SD; min–max)	3.08 (±2.81; 0–10)	2.71 (±2.42; 0–9)	0.140	4.00 (±3.11; 0–10)	3.62 (±2.89; 0–9)	0.311
**WHOQOL-OLD-8** M (±SD; min–max)	28.36 (±3.70; 19–37)	29.46 (±3.95; 20–37)	**0.009** *	27.54 (±4.05; 20–33)	27.92 (±4.37; 16–35)	0.490
**MoCA**: global M (±SD; min–max)	13.22 (±5.65; 3–24)	14.60 (±6.19; 2–25)	**0.005** *	15.35 (±4.41; 9–25)	16.73 (±6.00; 6–28)	0.076 **
**MoCA**: visuospatial/executive	1.34 (±1.08; 0–5)	1.30 (±1.11; 0–4)	0.748	2.15 (±1.35; 0–5)	2.12 (±1.34; 0–5)	0.948
**MoCA**: naming	1.64 (±1.06; 0–3)	1.89 (±1.10; 0–3)	0.077 **	1.73 (±0.96; 0–3)	1.81 (±1.02; 0–3)	0.635
**MoCA**: attention	2.74 (±1.94; 0–6)	3.02 (±1.91; 0–6)	0.218	3.38 (±1.75; 0–6)	3.46 (±1.94; 0–6)	0.967
**MoCA**: language	1.06 (±0.87 0–3)	1.28 (±1.11; 0–3)	0.202	1.46 (±0.95; 0–3)	1.77 (±0.95; 0–3)	0.194
**MoCA**: abstraction	0.74 (±0.78; 0–2)	0.82 (±0.85; 0–2)	0.682	0.73 (±0.78; 0–2)	0.88 (±0.71; 0–2)	0.360
**MoCA**: delayed recall	0.98 (±1.50; 0–5)	1.56 (±1.74; 0–5)	0.005 *	0.65 (±0.94; 0–3)	1.50 (±1.68; 0–5)	0.021 *
**MoCA**: orientation	4.72 (±1.39; (1–6)	4.74 (±1.52; 0–6)	0.667	5.27 (±1.04; 2–6)	5.19 (±0.94; 3–6)	0.439

**M**: mean; **SD:** standard deviation; **min**: minimum; **max**: maximum; * significant difference (*p* < 0.05; Wilcoxon test); ** marginally significant difference.

**Table 3:** The number of participants who improved, worsened, or maintained their clinical conditions after participating in each program.

	RT (*n* = 50)			CST (*n* = 26)			*p*
GDS-10	↑ 23 (46%)	↓ 21 (42%)	↔ 6 (12%)	↑ 7 (27%)	↓ 11 (42%)	↔ 8 (31%)	0.089 **
WHOQOL-OLD-8	↑ 26 (52%)	↓ 20 (40%)	↔ 4 (8%)	↑ 12 (46%)	↓ 11 (42%)	↔ 3 (12%)	0.830
MoCA	↑ 33 (66%)	↓ 13 (26%)	↔ 4 (8%)	↑ 14 (54%)	↓ 8 (31%)	↔ 4 (15%)	0.413

**↑** Number (%) of participants who improved after the intervention; **↓** number (%) of participants who worsened after the intervention; ↔ number (%) of participants without changes in their condition after the intervention; ** marginally significant differences (Chi-Square test).

## Data Availability

The data presented in this study are available on request from the corresponding author. The data are not publicly available due to ethical considerations regarding personal information and to respect what was written in the signed informed consent.
